# A Systematic Review of the Economic Burden of Type 2 Diabetes in Malaysia

**DOI:** 10.3390/ijerph17165723

**Published:** 2020-08-07

**Authors:** Kurubaran Ganasegeran, Chee Peng Hor, Mohd Fadzly Amar Jamil, Hong Chuan Loh, Juliana Mohd Noor, Norshahida Abdul Hamid, Purnima Devi Suppiah, Mohd Rizal Abdul Manaf, Alan Swee Hock Ch’ng, Irene Looi

**Affiliations:** 1 Clinical Research Center, Seberang Jaya Hospital, Ministry of Health Malaysia, Penang 13700, Malaysia; cheepengh@yahoo.com (C.P.H.); fadzly.crc@gmail.com (M.F.A.J.); lohhongchuan@gmail.com (H.C.L.); juliana_crc@yahoo.com (J.M.N.); norshahida.crc@gmail.com (N.A.H.); purnima.crc@gmail.com (P.D.S.); alanchng1978@gmail.com (A.S.H.C.); irenelooi@yahoo.com (I.L.); 2 Department of Medicine, Kepala Batas Hospital, Penang 13200, Malaysia; 3 Institute for Clinical Research, National Institutes of Health, Selangor 40170, Malaysia; 4 Department of Community Health, Faculty of Medicine, Universitiy Kebangsaan Malaysia, Kuala Lumpur 56000, Malaysia; 5 Medical Department, Seberang Jaya Hospital, Penang 13700, Malaysia

**Keywords:** diabetes, economic burden, cost of illness, healthcare costs, Malaysia

## Abstract

Diabetes causes significant disabilities, reduced quality of life and mortality that imposes huge economic burden on societies and governments worldwide. Malaysia suffers a high diabetes burden in Asia, but the magnitude of healthcare expenditures documented to aid national health policy decision-making is limited. This systematic review aimed to document the economic burden of diabetes in Malaysia, and identify the factors associated with cost burden and the methods used to evaluate costs. Studies conducted between 2000 and 2019 were retrieved using three international databases (PubMed, Scopus, EMBASE) and one local database (MyCite), as well as manual searches. Peer reviewed research articles in English and Malay on economic evaluations of adult type 2 diabetes conducted in Malaysia were included. The review was registered with PROSPERO (CRD42020151857), reported according to PRISMA and used a quality checklist adapted for cost of illness studies. Data were extracted using a data extraction sheet that included study characteristics, total costs, different costing methods and a scoring system to assess the quality of studies reviewed. The review identified twelve eligible studies that conducted cost evaluations of type 2 diabetes in Malaysia. Variation exists in the costs and methods used in these studies. For direct costs, four studies evaluated costs related to complications and drugs, and two studies were related to outpatient and inpatient costs each. Indirect and intangible costs were estimated in one study. Four studies estimated capital and recurrent costs. The estimated total annual cost of diabetes in Malaysia was approximately USD 600 million. Age, type of hospitals or health provider, length of inpatient stay and frequency of outpatient visits were significantly associated with costs. The most frequent epidemiological approach employed was prevalence-based (*n* = 10), while cost analysis was the most common costing approach used. The current review offers the first documented evidence on cost estimates of diabetes in Malaysia.

## 1. Introduction

Type 2 diabetes is a metabolic disease characterized by progressive loss of adequate β-cell insulin secretion, frequently because of insulin resistance [1]. The prevalence of diabetes has escalated rapidly amongst populations worldwide. Estimates from the International Diabetes Federation (IDF) reported that diabetes has affected 463 million people globally as of 2018, and this figure is projected to grow to almost 700 million people in 2045 [2]. Attributed to wide socioeconomic gaps, rapid urbanization, diet changes, increased sedentary lifestyles and population aging [3,4], the spatial distribution of diabetes prevalence rate reported globally was higher in low and middle income countries (LMICs) (13.5%) as compared to high income nations (10.4%). The Southeast Asia (SEA) region sustained the bulk of the total diabetes epidemic [5]. In 2018, Malaysia topped the Western Pacific region with a diabetes prevalence rate of 16.8%, affecting approximately 3.6 million people of the total adult population [6].

People with diabetes are predisposed to macrovascular complications such as cardiovascular, cerebrovascular, and peripheral vascular diseases, as well as microvascular complications like retinopathies, nephropathies and neuropathies [7,8,9]. These complications accelerate significant premature mortalities, loss of productivity and poor quality of life [10,11]. The debilitating nature of the disease exacerbates an excruciatingly costly burden to patients, caregivers and healthcare systems across countries worldwide [3,11]. Global healthcare expenditure for the management of diabetes and its complications amongst diabetes people aged between 18 to 99 years old was estimated to be USD 850 billion in 2017, and this figure is projected to rise by 7% to USD 958 billion in 2045 [12]. Expenditure for the management of diabetes within the Western Pacific region was the second highest (USD 62.2 billion) after the North American and Caribbean regions (USD 324.5 billion) [2,13].

While the impact of diabetes to society is notable, the economic costs borne by healthcare systems and governments are hugely substantial. The burden of diabetes amplifies significant direct and indirect consequences to patients. While the direct costs deal with healthcare resource consumption such as medications, outpatient and inpatient costs, the indirect costs largely encompass monetary losses caused by disabilities and deaths, travel expenses, nutrition costs, productivity and income loss [3]. Financing of healthcare delivery in Malaysia is highly subsidized by the government, although Malaysia has a parallel healthcare delivery system from both private and public healthcare entities. In 2018, budget allocation to the Ministry of Health (MOH) Malaysia was MYR 28.7 billion (equivalent to USD 7.1 billion), accounting to approximately 9% of the total budget spending and government revenues [14].

It is crucial to understand resource utilization of the allocated budget to manage the diabetes burden and its complications. Succinctly, Malaysia has commenced using pharmacoeconomic evidences for regulatory assessments of new drugs [15]. These targeted aims require specific application of common costing techniques, especially cost analysis, cost-of-illness analysis and cost–benefit analysis to yield appropriate economic information for comprehensive knowledge to stakeholders, health systems and governments for policy drafting [16]. It should be noted that significant variations in costing methodologies used for different study aims and objectives across different countries and settings make it difficult for economic advocates to compare cost estimates or to extrapolate yielded estimates to a nationally or internationally representative level [17,18]. However, the development and improvement of cost-effective diabetes management depends solely on cost identification. Systematic reviews assist to critically summarize the findings, discuss the methods used and identify gaps in the literature. The alarming rate of type 2 diabetes and its complications in Malaysia has driven this study to systematically appraise, synthesize and analyze the costs of type 2 diabetes mellitus in Malaysia. The findings, the first in the country, document the economic burden and its associated factors affecting type 2 diabetes in Malaysia.

### 1.1. Overview of Health Economic Evaluation

Economic assessments require identification and measurement of costs related to the illness, treatment or program under evaluation. Commonly identified costing components within the healthcare system are categorized into direct, indirect, intangible, capital, recurrent and per diem costs [11,16,19,20] as described in Section 1.1.1, Section 1.1.2, Section 1.1.3, Section 1.1.4, Section 1.1.5 and Section 1.1.6.

#### 1.1.1. Direct Costs

Direct costs are correlated with resources used to manage an illness. Direct costs are regarded as the primary cost of healthcare programs and often include expenditure for medical care or treatment of the disease. Directs costs are typically subdivided into direct medical costs (including health service costs such as hospitalizations, outpatient follow-ups, medications, laboratory investigations, medical supplies, emergency medicine services and traditional medicine services) and direct nonmedical costs (costs incurred by patients, family members or relatives, such as travel and meal expenses while traveling to the healthcare facility).

#### 1.1.2. Indirect Costs

Indirect costs are secondary costs associated with paid or unpaid productive activities. Such costs can emerge when treatment is confined within the hospital or home, causing the expenses to be incurred by patients or their families due to productivity loss as a result of significant morbidity, disability or mortality.

#### 1.1.3. Intangible Costs

Intangible costs are nonquantifiable costs. These include social, emotional and human costs that do not convert to money, such as pain, suffering or loss of quality of life.

#### 1.1.4. Capital Costs

Capital costs are costs that evaluate buildings, land, vehicles and equipment that require special consideration. Such costs arise at a single point of time, but the assets are used over a period of time.

#### 1.1.5. Recurrent Costs

Recurrent costs include staff emoluments that are involved in the management of the disease, overhead costs such as operation and maintenance of the building, equipment or vehicles. It also includes consumables, drugs, and investigation costs, such as laboratory, radiological, and ECG tests.

#### 1.1.6. Per Diem Costs

Per diem costs refer to per bed-day costs for patients admitted into the hospital, which include administrative, food and kitchen service costs. It measures resource consumption for each patient but excludes costs directly related to medical care, such as drugs or special consumable items.

## 2. Materials and Methods

A comprehensive systematic search of the literature regarding economic burden and cost evaluation of type 2 diabetes mellitus in Malaysia was conducted between September and October 2019 following the Preferred Reporting Items for Systematic Review and Meta-Analyses (PRISMA) guidelines [21]. This systematic review was registered with the international prospective register of systematic reviews registration (PROSPERO) CRD42020151857 and the National Medical Research Registry (NMRR) of Malaysia (NMRR-19-2826-51004).

### 2.1. Data Sources and Search Strategy

Major English databases that include PubMed, Scopus and EMBASE, and the Malaysian only database, Malaysian Citation Index (MyCite), were explored to identify relevant studies reporting cost evaluation of diabetes and its complications among adults in Malaysia between 1 January 2000 and 31 August 2019. In addition, a manual search of the reference lists in the articles included for the review was used to identify further eligible articles. Only original research articles in English and Malay from peer-reviewed publications were included.

### 2.2. Search Terms

Search terms and their combinations are presented in Table 1. A two-tier screening process was conducted to retrieve relevant articles by two trained independent reviewers. One primary term associated with “Malaysia” in combination with one term associated with diabetes and its complications, and one term associated with cost evaluation were searched. Boolean operators AND or OR were utilized during combinatory keywords search. Firstly, the titles of the articles were screened under the following terms: (“Malaysia” OR “Malaysian”) AND “diabet *” AND (“complications” OR “stroke” OR “myocardial infarction” OR “nephropathy” OR “neuropathy” OR “retinopathy” OR “chronic kidney disease” OR “chronic renal disease”) AND (“cost” OR “expenditure” OR “economic burden” OR “expenses” OR “cost analysis”). The term “diabet *” was truncated for the extension of terms “diabetes,” “diabetic,” “diabetics,” and “diabetology.” Secondly, two reviewers independently reviewed the abstracts and, if necessary, read the full texts of all the articles that were selected based on the inclusion criteria. Any disagreement between the two reviewers was finalized upon discussion with a third reviewer, when necessary.

### 2.3. Inclusion Criteria

Articles were included if they provided original research findings related to costs (direct, indirect, intangible, capital and recurrent) of type 2 diabetes mellitus and its complications in Malaysia following the Population, Intervention, Comparison, Outcome, Study (PICOS) type approach for systematic reviews. Population considered were people with type 2 diabetes mellitus in Malaysia; the contexts of interest were hospitals, primary healthcare clinics and home settings; direct, indirect, intangible, capital and recurrent costs from the healthcare (provider) and individuals’ perspective comprised the outcomes; and relevant study designs were observational (cross-sectional or surveys), randomized controlled trials (RCTs) and modeling studies.

### 2.4. Critical Review and Quality of Studies

Articles that reported on the economic burden of diabetes using both quantitative and qualitative methods were included to extract data related to costs. An extraction table using an Excel spreadsheet was developed for the summarized data retrieval. In the first part, variables such as year of publication, study design, setting and data sources were retrieved. The second part retrieved cost-related data. Historical conversion rates were based on average conversion rate per year for that particular financial year (MYR to USD). The third part included items related to the evaluation of quality and methodological soundness of cost of illness (COI) studies. We utilized a checklist developed by Drummond et al. [22] for economic evaluation consisting of 10 items, which was adapted for use by previous studies [23,24,25]. Equal weight was assigned to each of the items with a final score being the sum of the ten individual items. Two reviewers independently assessed each of the articles included in this review. Any disagreement between reviewers at that stage was brought forward for discussion with a third reviewer and with reference to the COI study checklist until consensus was reached.

## 3. Results

### 3.1. Search Results

**Figure 1:**
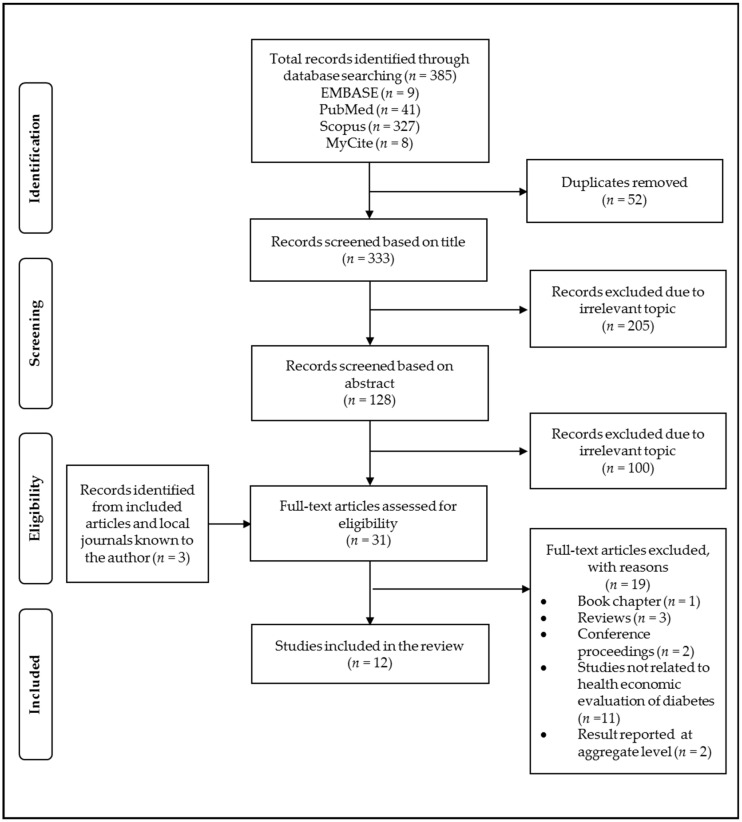
Preferred Reporting Items for Systematic Review and Meta-Analyses (PRISMA) flowchart of the study selection process.

The initial search identified 385 records. From these, 52 records were duplicates and were removed. Of the remaining 333 records, 305 were excluded through screening of titles and abstracts, leaving 28 articles for full-text review. These 28 articles were screened manually and 3 additional articles known to authors were included. After assessing the full-text articles, 19 were excluded. A total of 12 studies were selected for final inclusion into this systematic review (Figure 1).

### 3.2. General Characteristics of the Studies

Table 2 shows the general characteristics of the studies included. Ten studies were observational cross-sectional studies, one study was a modeling study and another one was a randomized-controlled trial. Two-thirds (*n* = 8) of the studies were conducted by local researchers in healthcare facilities in Malaysia [26,27,28,29,30,31,32,33]. Six international studies were initially identified during the selection process. However, only four multicountry studies (three from Asia and one global study) were selected as these studies included Malaysia in their cost estimate analyses [34,35,36,37]. Another two studies that reported data at the aggregate level [38,39] were excluded in our analysis as they did not provide detailed country-specific cost estimates of diabetes. The remaining studies were region specific that included one study from the Peninsular Malaysia [33], one study in the state of Penang [30], two studies in the state of Kelantan [26,29], one study in the state of Selangor [28] and two studies in the Federal Territories of Kuala Lumpur [27,32]. The majority of the studies had sample size that ranged between 100 to 3500 (six studies) [26,28,29,32,33,36], three studies had sample sizes of less than 100 [27,30,34] and another three studies had no sample sizes calculated, as they used secondary data [31,35,37]. Only two studies generalized the estimated cost burden of diabetes for the national population using secondary data sources [31,37]. Forty-two percent of the studies estimated the cost of managing diabetes complications, while 33% estimated the general cost of diabetes. Three studies (25%) estimated the cost of specific drugs for the treatment of diabetes and its complications.

### 3.3. Epidemiological Approaches, Types and Perspectives of Costs

Most common epidemiological approaches used in health economic evaluations are either the prevalence-based approach or the incidence-based approach [40]. The prevalence-based approach is applied to estimate the economic burden that is attributed to the prevalence of cases over a specific period of time, while the incidence-based approach involves analyzing the cost of diabetes over a given period of time [41]. In this review, most studies (*n* = 10) estimated the cost of diabetes using the prevalence-based approach [26,27,28,29,30,31,32,33,36,37], while two studies used an incidence-based approach for cost estimation [34,35].

A global study estimated the cost of diabetes by using country-by-country expenditures [37]. Six studies measured direct medical costs [30,31,33,34,35,36]. One study measured indirect and intangible costs [29]. Four studies estimated capital and recurrent costs [26,27,28,32].

Various perspectives are used when analyzing health economic studies. The common ones include patient’s perspective, such as out-of-pocket payments, employer’s perspective like loss of productivity; health system perspective, like evaluating costs of hospital and primary healthcare (clinic services); government’s perspective, such as estimating the infrastructure or health promotion/support programs; and lastly, the societal perspective to evaluate income loss during the care for the sick [25,42]. In this review, the bulk of studies (*n* = 7) reported cost estimates from the healthcare system (provider) perspective [27,31,32,34,35,36,37]. Four studies reported cost estimates from the government’s perspective [26,28,30,33], and only one study took a societal perspective [29].

### 3.4. Variations in Costing Approaches

The single global study that estimated country-by-country expenditures for diabetes utilized the sum-all medical approach [37]. Cost analysis approach was the most common method of costing used in estimating direct [31,33,34], and capital and recurrent [27,28] costs of diabetes in Malaysia. Another two studies that estimated direct costs [30,35] and one study that evaluated capital and recurrent costs [26] utilized the cost-effectiveness analysis approach. Cost-of-illness approach was utilized in one study that evaluated capital and recurrent costs [32]. A single direct cost evaluation study that evaluated inpatient treatment of diabetes among patients being insured or uninsured adopted the per diem approach [36]. The only indirect and intangible cost evaluation study adopted the cost–benefit analysis approach by using the willingness to pay (WTP), contingent valuation (CV) technique [29].

### 3.5. Costing Components

Of the six studies that reported the costing components to estimate direct costs, two studies estimated the costs for outpatient follow-up visits, two looked into inpatient costs, three reported drug costs, including drugs for diabetes complications and another three reported on estimated costs of managing diabetes complications.

Four studies that evaluated capital and recurrent costs also reported the respective costing components. Capital costs that included buildings, equipment and vehicles were reported for all studies. Similarly, for recurrent costs, one study estimated operation and maintenance costs for building, one study reported operation and maintenance costs for vehicle, one study reported on training cost, three studies reported staff emoluments, two studies reported on investigation costs, three studies reported on drug costs, two studies reported on consumables costs and one study reported administrative, food and kitchen service costs.

### 3.6. National Economic Burden of Type 2 Diabetes

Of the twelve studies identified in this review, only two studies [31,37] estimated the overall healthcare expenditure of type 2 diabetes in Malaysia. While a multicountry study that estimated the total healthcare expenditure for the treatment of diabetes in Malaysia in 2010 was USD 600,407,750 [37], a local study found that the estimated cost of diabetes management in 2011 was approximately USD 667,102,680 with the government bearing a sum of USD 457,815,570 (68.6%) of the total cost expenditure [31] (based on average conversion rate for financial year of 2011; 1 USD = MYR 3.06).

### 3.7. Direct Costs

Four out of six studies that estimated direct costs were related to diabetes complications. One study reported that the management of end stage renal disease (ESRD)/nephropathy per patient per month on hemodialysis accounted for USD 795 and peritoneal dialysis accounted for USD 787 [34]. Another study estimated costs per patient per year for hospitalized diabetic patients with anemia and ESRD as USD 364.50 [33,35], and subsequently reported that the total costs for a diabetic patient to undergo dialysis accounted for USD 19,054 per year, while the cost of renal transplant in the first year and postindexed year accounted for USD 70,022 and USD 14,111, respectively. Mustapha et al. [31] estimated the costs of diabetes complications per patient per year for myocardial infarction (USD 1575.21), stroke (USD 1747.87), retinopathy (USD 156.64), foot amputation (USD 1804.77), heart failure (USD 1747.87) and ophthalmic procedures (USD 1573.58). Drug costs were reported in four studies. Seng et al. [34] reported that management of ESRD in diabetes patients using Losartan over 3.5 years was USD 1888. The mean cost of oral antidiabetic agents per patient per month was estimated to be USD 5.35 at baseline, USD 2.94 at 3 months follow-up and USD 3.05 at 6 months follow-up [30], while drug costs of a diabetic patient per year was estimated to be USD 452.49, and USD 396.07 for diabetic patients with anemia [33]. Drug cost of diabetic patient with hypertension given amlodipine was USD 322 and irbesartan was USD 258 per patient per year [35]. Outpatient diabetes care per patient per year ranged between USD 52.80 and USD 150.10 according to two studies [31,35]. Two other studies reported inpatient costs, ranged between USD 264.98 and USD 6439.87 per patient per year [33,36] (Table 3).

### 3.8. Indirect and Intangible Costs

Only one study measured the indirect and intangible costs via a cost–benefit analysis approach for a diabetes self-management program [29]. Through willingness to pay approach, this study elicited that the total provider cost for the program was USD 9558.65, while patients’ willingness to pay for the program was USD 11,570.24, resulting in a net benefit of USD 2011.59.

### 3.9. Capital Costs

Four studies evaluated capital costs for diabetes management. All of these studies evaluated costs for buildings while three of the studies evaluated costs for equipment. Only one study estimated cost for a vehicle. For outpatient health clinic visits, average total capital costs per year ranged between USD 6.50 [26] and USD 55.33 [28]. For outpatient hospital visits, average total capital costs were USD 48.78 per year for consultations without specialists and USD 97.68 per year for consultations with specialists [28]. In the hospital setting, cost for treating patients with diabetic foot per day was estimated to be USD 5.02 [27]. Total capital costs for inpatients ranged between USD 48.67 [32] and USD 223.42 [28], whereas total capital costs for outpatients at the hospital setting ranged between USD 34.60 [32] and USD 94.82 [28] (Table 4).

### 3.10. Recurrent Costs

Recurrent costs were evaluated in four studies [26,27,28,32]. Estimation for recurrent costs included the following costing components: operation and maintenance of building, equipment and vehicle, training, emolument, tests/investigations, drugs, consumables, administration and food and kitchen services. For outpatient health clinic visits, average total recurrent costs per year ranged between USD 57.30 [28] and USD 245.73 [26]. For outpatient hospital visits, average total capital costs were USD 48.78 per year for consultations without specialists and USD 97.68 per year for consultations with specialists [28]. In the hospital setting, recurrent cost for treating patients with diabetic foot per day was estimated to be USD 129.74 [27]. Total inpatient recurrent costs ranged between USD 166.05 [32] and USD 395.62 [28], whereas total outpatient recurrent costs at hospital settings ranged between USD 118.07 [32] and USD 147.47 [28] (Table 5).

### 3.11. Costs Associated Factors

Of the twelve studies in this review, two studies described the factors associated with costs by using univariate analyses only [29,36], two studies used both univariate and multivariate analyses [26,28] and one study used univariate and propensity score matching analyses [33]. Results of the univariate analyses found that education level, income, duration of diabetes, clinical profiles, comorbidities and complications were associated with costs. At the multivariate level, age, type of hospitals or health provider, length of inpatient stay and frequency visits of outpatients were significantly associated with costs. The multivariate model concluded that length of stay was the most significant factor associated with inpatient costs, whereas frequency of visits and type of healthcare provider (health facility with specialists or without specialists) highly influenced the costs for outpatient visits [28].

### 3.12. Methodological Quality of the Economic Evaluation Studies

The results were clearly presented in most studies and consistently well reported in relation to the methods adopted (Table 6). For six studies, responses to seven of the ten items were “yes.” All studies scored “yes” on items 1 and 6. However, the study by Zhang et al. [37] did not specify accurately whether it refers to diabetes type 1, type 2 or both. We assumed it to be diabetes type 2 based on article discussion and references that mostly referred to diabetes type 2. Epidemiological sources of most studies were clearly described. The majority of the studies included in this review discussed the limitations regarding the methodologies employed in calculating costs. A common weakness discussed was that studies collected data from single setting or insufficient sample size, thus disallowing the findings to be extrapolated for national estimates.

Costs were discounted in four studies. In all studies, the discount rate was 3% [26,29,34,35]. Most studies with a time horizon less than one or two years did not discount costs. Sensitivity analysis was conducted in four studies. The analysis was conducted by using deterministic (one-way deterministic sensitivity analysis) and probabilistic (second-order Monte Carlo simulation analysis) approaches in one study [35], while another study conducted sensitivity analysis by establishing a range of highest and lowest likely costs of diabetes using unit costs of treatment from the literature [31]. Al-Haddad et al. [29] conducted sensitivity analysis based on the assumption that a diabetes self-management program would result in the prevention of complications, while Rohana et al. [26] performed sensitivity analysis with variable discount rates of 0, 5 and 7% in comparison to the standard 3% of the study discount rate. 

## 4. Discussion

This systematic review is the first to compile all available reports on economic analyses of type 2 diabetes in Malaysia between 2000 and 2019. This study contributes to the understanding of type 2 diabetes cost burden in Malaysia and the methods used to evaluate these costs. The review records existing evidence in the literature on the overall costs of managing diabetes in Malaysia, the different costing components evaluated, the perspectives used and the factors that influenced cost estimates. A total of twelve studies were included: eight local studies, three Asian regional studies and one global study that reported country-specific estimates. Two studies attempted to generalize the cost estimates nationwide, while the remaining studies focused on cost estimation either from a single-center or multisite within certain states in the country.

### 4.1. Overall Cost Burden of Type 2 Diabetes in Malaysia

Type 2 diabetes has been a growing public health concern in Malaysia over the past two decades. The prevalence progressively increased from 8.3% in 1996 to 14.9% in 2006 and 17.5% in 2015 [44,45], with the recent figure rising up to 20% in 2019, whereby nearly half were undiagnosed [46]. The prevalence is projected to double up to 31.3% of the 36.02 million population by 2025 [47]. In 2010 alone, an estimated USD 600 million was spent on diabetes-related healthcare in the country, accounting for approximately 16% of the overall national healthcare budget [37]. Our estimate was within the range of the total annual costs, between USD 141.6 million and USD 174 billion, as estimated by Ng et al. [48] in their global systematic review. There were reports estimating economic costs or cost of illness for individual countries, but systematic reviews of national economic burden for type 2 diabetes remained limited. In neighboring countries such as Singapore, the total economic cost of type 2 diabetes for the country’s working-age population, nearly 140,000 persons, was estimated to be USD 787 million in 2010, accounting for 0.35% of the country’s gross domestic product [49]. The direct medical costs and complications in China were estimated to be USD 26 billion in 2007 [50], while the total estimated cost of diabetes was USD 174 billion in the United States of America in 2007 [51] and USD 31.9 billion in India in 2010 [52]. The selection of cost-estimation methodology was largely driven by data availability and perspectives of each study, which in turn influenced the magnitude of cost estimates. As compared to high income countries, low and middle income countries (LMICs) require more large-scale national studies with robust methodological economic analyses to evaluate consistencies [11,53].

### 4.2. Perspectives of Costs Burden

The Malaysian healthcare system is a two-tier system comprising public and private sectors, with voluntary private medical insurance coverage made available for the population. The public healthcare system is substantially subsidized by the government and the private healthcare system is accessible voluntarily by the population, mainly funded from out-of-pocket expenditure and insurance coverage. The medical card covers hospitalization and short-term follow ups after discharge and does not provide long-term outpatient care for insured persons. In 2017, the government subsidized 43.1% of the total national health expenditure (USD 13.6 billion), with 37.6% borne by the population via out-of-pocket expenditure. Nearly half (49%) or USD 4.8 billion of the out-of-pocket health expenditures were consumed by private hospitals, followed by private medical clinics (22%) and private pharmaceutical services (14%) [54]. Private insurance enterprises contributed to 7% of total healthcare expenditures in the same year [54] and only one-third of the population possessed private medical insurance coverage [55].

The diabetes care model in Malaysia continues to evolve. Up to 80% of patients with diabetes sought care at health clinics (56%) or hospitals (24.6%) financed by the Ministry of Health, followed by private clinics (15.0%), private hospitals (2.5%) and self-medication (1.7%) [56]. As such, the cost estimated by studies conducted based on public healthcare settings compiled in this systematic review remained conservative and underestimated the out-of-pocket expenditures by the Malaysian population. More studies on out-of-pocket expenditures on diabetes care and hospitalization in private hospitals are needed to enable development of a future diabetes-care economic model for Malaysia.

Burden from patients and societal perspective, such as loss of productivity due to diabetes-related complications and disability, needs to be studied extensively. Families with members who have diabetes bear a disproportionate share of burden in terms of reduced earnings and higher out-of-pocket expenditures [57]. Studies on intangible costs to evaluate reduced quality of life that impose greater burden to the society are frequently omitted from the economic burden estimate and need to be accelerated.

### 4.3. Costing Components

Half of the economic burden studies in this review focused on diabetes-related renal diseases. There was only one national study that estimated the direct medical cost for selected macrovascular and microvascular complications. This study was based on the incidence rates from the UKPDS trial rather than incidence rates in Malaysia, which were lacking. Besides, additional costing studies on antidiabetic medications such as injectable therapies and newer oral agents, as well as medications for comorbidities for patients with diabetes, are needed to provide better estimates of the direct medical cost. Capital and recurrent cost estimates provide additional insights into planning of resource distribution. In the study by Ezat et al. [28], the total recurrent cost per patient admission and outpatient care were higher for hospitals without specialists as compared to hospitals with specialists, yet being attributable to higher operation and maintenance cost of buildings, equipment and vehicles, staff emoluments, medication prescriptions, and food and kitchen services. Similar studies are needed to further explore and understand the pattern of these cost estimates.

Variations in cost estimation models also pose substantial challenges in the quantification of economic burden. For instance, annual direct medical costs for hemodialysis estimated by Seng et al. [34] was USD 9444, and USD 19,054 by Annemans et al. [35], revealing an approximate 50% discrepancy. Among the 12 studies, the study by Annemans et al. [35] was one of the studies that had major assumptions tested in a sensitivity analysis, to verify their estimation.

### 4.4. Factors Influencing Costs Burden

Various factors have been found to be associated with direct costs of diabetes care, which include education, income, number of diabetes-related complications, duration of diabetes, treatment options, hospitalizations and surgery [11]. In our study, results of the univariate analyses found that education level, income, duration of diabetes, clinical profiles, comorbidities and complications were associated with costs. At the multivariate level, the age, type of hospital or health provider, length of inpatient stay and frequency visits of outpatients were significantly associated with costs. The multivariate model concluded that length of stay was the most significant factor associated with inpatient costs, whereas frequency of visits and type of healthcare provider (health facility with specialists or without specialists) highly influenced the costs for outpatient visits. Patients with longer duration of diabetes develop proportionately more complications and the condition is further exacerbated by poorly controlled diabetes, hence direct medical cost increases. The higher cost is associated with a higher level of hospital care and length of hospital stay for treatment of complications [3]. In addition, commercial medical insurance was found to be associated with higher hospitalization and treatment cost in China, as compared to those without insurance coverage [3,58]. This has yet to be evaluated in Malaysia.

### 4.5. Strengths and Limitations

The epidemiological approaches used in most studies were prevalence-based, which are mostly suited to measure costs related to chronic diseases such as diabetes. This finding was consistent with previous studies [25,48,53]. Prevalence-based studies measure disease attributable cost that occur concurrently with prevalent cases over a specified time period, hence are highly reliable to assess the total economic burden of a disease [42]. However, prevalence-based studies include a cross-section of cases that may reflect costs at varying stages of a disease, including those which are not amenable to clinical interventions. With this limitation, prevalence-based approaches are less reliable to measure potential savings from preventive interventions [42].

The current available literature on costs of diabetes and its related complications in this study provided a fragmented picture focusing on cost estimation from limited public healthcare facilities rather than the entire healthcare system in the country. The cost variation between rural and urban settings should also be addressed. Data biases and limitations in terms of contextualization of results also pose concerns in systematic reviews of economic burden of diabetes worldwide [18,53,59]. There is a need to develop a robust methodology to conduct comprehensive studies with generalized cost estimation to inform policy-making decisions [53].

## 5. Conclusions

The national economic burden for type 2 diabetes was estimated to be USD 600 million, which was an underestimate based on limited studies. This underscores the need to develop comprehensive diabetes cost models to understand the economic burden of type 2 diabetes, which could form a platform to guide efficient resource distribution and planning of diabetes care in the population.

## Figures and Tables

**Table 1:** Search terms.

		Combined with (Individually)	Combined with (Individually)
Malaysia	diabet *	complications	cost
Malaysian		stroke	expenditure
		myocardial infarction	economic burden
		nephropathy	expenses
		neuropathy	cost analysis
		retinopathy	
		chronic kidney disease	
		chronic renal disease	

* Term truncated.

**Table 2:** General characteristics of the studies included in the review.

Ref.	Author	Year	Study Design	Region	Setting	Sample Size	Study Period	Diabetes Complications	Data Source
[33]	Azmi et al.	2018	CS	Peninsular Malaysia	20 health clinics	514	Oct.–Dec. 2015	Anemia, chronic kidney disease	Public clinic patient data and secondary data
[31]	Mustapha et al.	2017	CS	National	*	Not available	2011	Myocardial infarction, stroke, retinopathy, foot amputation, heart failure, nephropathy and cataract extraction	Secondary data include NHMS, UKPDS, Malaysian National Renal Registry, MOH, MIMS, MMA schedule of fees 2002; clinical expert opinions
[32]	Ismail et al.	2017	CS	Federal Territory (Kuala Lumpur)	UKM Medical Center, Malaysia	217 inpatients, 3214 outpatient visits	2013	NA	Hospital inpatient data and financial administrative data
[30]	Lim et al.	2012	CS	State (Penang)	Endocrine Clinic, Penang General Hospital	80	Sept. 2008–Dec. 2009	NA	Public hospital patient data
[37]	Zhang et al. **	2010	CS	Global	*	Not available	2010 and 2030 (projection)	NA	Secondary data include WHO published data, country-by-country estimates, UN population data
[36]	Fiebert et al.	2010	CS	Asia	UKM Medical Centre, Malaysia	100	2005–2008	NA	Tertiary hospital inpatient medical expenditures and insurance data
[29]	Al-Haddad et al.	2010	CS	State (Kelantan)	Health Center of University Science Malaysia	135	Aug. 2005–Jan. 2006	End-stage renal disease	Hospital human resource department data and survey
[27]	Amin et al.	2009	CS	Federal Territory (Kuala Lumpur)	UKM Medical Centre, Malaysia	54	2006	Diabetic foot, chronic kidney disease	Hospital patient data
[28]	Ezat et al.	2009	CS	State (Selangor)	One government hospital with specialists, two government hospitals without specialists and five health clinics	361 (226 outpatients, 135 inpatients)	Sept.–Nov. 2015	NA	Patients, hospitals and clinics using economic evaluation forms and guided questionnaires
[35]	Annemans et al.	2008	MS	Asia	*	Not available	2004	End-stage renal disease, nephropathy	Secondary data include National Dialysis and Transplant Registry, published peer-reviewed data, average costs in the private and NGO sectors, MMA schedule of fees, 2002
[26]	Rohana et al.	2007	CS	State (Kelantan)	Two government health clinics	300 (155 without FMS, 145 with FMS)	Aug. 2005–May 2006	NA	Public clinics
[34]	Seng et al.	2005	RCT	Asia	*	21	2004	End-stage renal disease, nephropathy	Public hospital patient data

CS = cross-sectional study, RCT = randomized controlled trial, MS = modeling study, FMS = Family Medicine Specialists, WHO = World Health Organization, UN = United Nations, NHMS = National Health and Morbidity Survey, UKPDS = UK Prospective Diabetes Study, MOH = Ministry of Health, MMA = Malaysian Medical Association, NA = Not Applicable; * studies that did not specify clear indicators on the setting such as public or private healthcare facilities, hospitals or clinics; ** the study did not clearly state whether it refers to diabetes type 1, type 2 or both (assumed to be diabetes type 2 based on article references that mostly referred to diabetes type 2).

**Table 3:** The direct medical costs for type 2 diabetes.

Ref.	Author	Year	Data Source	Direct Medical Costs
[33]	Azmi et al.	2018	Case report forms, price resources obtained from published literature, publicly available information to match against resource utilization	Hospitalizations of diabetes patients per year with anemia 1034.50 (264.98), without anemia 732.42 (187.61), with anemia and ESRD 1423 (364.50). Drug costs of diabetes patients per year with anemia 1546.26 (396.07), without anemia 1766.52 (452.49). Outpatient clinic visits of diabetes patients per year with anemia 215.37 (55.17), without anemia 206.15 (52.80).
[31]	Mustapha et al.	2017	Epidemiological data from UKPDS 35, NHMS 2011 Malaysian National Renal Registry. Resource use quantities from MOH, clinical experts, expert opinion. Unit costs from MOH, MIMS 127th Ed., 2011, MMA schedule of fees 4th Ed., 2002, clinical experts	Outpatient diabetes care per patient per year 459 (150.10); hospitalization costs per patient per year for myocardial infarction 4817 (1575.21), stroke 5345 (1747.87), retinopathy 479 (156.64), foot amputation 5519 (1804.77), heart failure 5345 (1747.87), ophthalmic procedures 4812 (1573.58).
[30]	Lim et al.	2012	Patient medical records	Mean cost of oral antidiabetic agents per month for baseline 18.85 (5.35), 3 months 10.35 (2.94), 6 months 10.63 (3.05). The cost was calculated from switching of generic precombined medications to innovator medications.
[36]	Fiebert et al.	2010	Inpatient medical expenditures, hospital records and patient’s insurance	Inpatient cost with insured per patient per year 893.80 (268.33) and uninsured patient 21,451.20 (6439.87).
[35]	Annemans et al.	2008	National Dialysis and Transplant Registry, published peer-reviewed data, average costs in the private and NGO sectors, MMA schedule of fees, 2002	Drug cost of diabetes patient with hypertension per year given amlodipine 1223.60 (322) and irbesartan 980.40 (258). Cost of a diabetes patient on dialysis per year 72,405.20 (19,054). Cost of renal transplant of diabetes patient in the first year 266,083.60 (70,022) and cost postindex year of transplanted patient 53,621.80 (14,111).
[34]	Seng et al.	2005	Results from the RENAAL study [43] and estimates of Malaysian dialysis costs from 44 hemodialysis and 11 continuous peritoneal dialysis centers	Management of ESRD/nephropathy (per patient per month) for hemodialysis 3021.00 (795), peritoneal dialysis 2990.60 (787). Management of ESRD with losartan at 3.5 years follow-up per patient 7174.40 (1888)

Cost values are reported as MYR (USD average conversion rate per year of that particular financial year); ESRD = end stage renal disease; RENAAL = Reduction of Endpoints in NIDDM with the Angiotensin II Antagonist Losartan

**Table 4:** Costing items, estimates per person and total costs in studies reporting capital costs of diabetes individuals.

Ref.	Author	Year	Building	Equipment	Vehicle	Total Capital Costs
[32]	Ismail et al.	2017	Cost inpatient per episode of care 153.26 (48.67), outpatient per visit 108.97 (34.60)	NA	NA	Cost inpatient per episode of care 153.26 (48.67), outpatient per visit 108.97 (34.60)
[27]	Amin et al.	2009	Cost per day 12.52 (3.42)	Cost per day 5.86 (1.60)	NA	Cost per day 18.38 (5.02)
[28]	Ezat et al.	2009	Cost per admission for hospital with specialist 802.99 (211.31), without specialist 393.21 (103.72) Cost outpatient per year for hospital with specialist 340.63 (89.64), without specialist 174.28 (45.86), health clinic 211.73 (55.72)	Cost per admission for hospital with specialist 69.24 (18.22) without specialist 36.72 (9.66) Cost outpatient per year for hospital with specialist 30.55 (8.04) without specialist 11.07 (2.91), health clinic 4.28 (1.13)	NA	Cost per admission for hospital with specialist 872.23 (229.56) without specialist 429.93 (113.14) Cost outpatient per year for hospital with specialist 371.18 (97.85), without specialist 185.35 (48.78), health clinic 216.01 (56.84)
[26]	Rohana et al.	2007	Cost per year for clinic with FMS 17.56 (4.79), without FMS 7.17 (1.96)	Cost per year for clinic with FMS 7.74 (2.11) without FMS 4.43 (1.21)	Cost per year for clinic with FMS 8.67 (2.36) without FMS 3.79 (1.03)	Cost per year for clinic with FMS 33.99 (9.27) without FMS 15.39 (4.20)

Cost values are reported as MYR (USD average conversion rate per year of that particular financial year); NA–not available.

**Table 5:** Costing costs, estimates per person and total costs in studies reporting recurrent costs of diabetes individuals.

Ref.	Author	Year	Operation and Maintenance of Building	Operation and Maintenance of Equipment	Operation and Maintenance of Vehicle	Training	Emolument	Test/Investigations	Drug	Consumables	Administration	Food and Kitchen Services	Total Recurrent Costs
[32]	Ismail et al.	2017	NA	NA	NA	NA	Cost inpatient per episode 144.24 (45.81), outpatient per visit 102.56 (32.57)	NA	Cost inpatient per episode 378.63(120.24), outpatient per visit 269.23 (85.50)		NA	Cost inpatient per episode 522.87 (166.05), outpatient per visit 371.79 (118.07)	NA
[27]	Amin et al.	2009	Cost per day 50.61 (13.81)	NA	NA	NA	NA	NA	NA	NA	NA	NA	Cost per day 475.64 (129.74)
[28]	Ezat et al.	2009	Cost per admission for hospital with specialist 307.79 (80.00), without specialist 402.93 (106.03) Cost outpatient per year for hospital with specialist 89.12 (23.45), without specialist 101.39 (26.68), health clinic 12.74 (3.35)			NA	Cost per admission for hospital with specialist 329.21 (86.63), without specialist 690.69 (181.76) Cost outpatient per year for hospital with specialist 138.19 (36.37), without specialist 261.34 (68.77), health clinic 96.37 (25.36)	Cost per admission for hospital with specialist 41.22 (10.85), without specialist 36.30 (9.55) Cost outpatient per year for hospital with specialist 18.20 (4.79), without specialist 10.58 (2.78), health clinic 8.06 (2.12)	Cost per admission for hospital with specialist 249.59 (65.68), without specialist 298.98 (78.68) Cost outpatient per year for hospital with specialist 102.47 (26.97), without specialist 122.07 (32.12), health clinic 60.34 (15.88)	NA	Cost per admission for hospital with specialist 121.23 (31.90), without specialist 81.89 (21.55) Cost outpatient per year for hospital with specialist 53.52 (14.08), without specialist 30.33 (7.89), health clinic 40.23 (10.59)	Cost per admission for hospital with specialist 30.08 (7.92), without specialist 33.63 (8.85)	Cost per admission for hospital with specialist 1078.77 (283.88), without specialist 1544.51 (406.45) Cost outpatient per year for hospital with specialist 401.51 (105.66), without specialist 525.71 (138.34), health clinic 217.74 (57.30)
[26]	Rohana et al.	2007	Cost per year for clinic with FMS 10.01 (2.73), without FMS 14.37 (4.00)	Cost per year for clinic with FMS 20.02 (5.46), without FMS 13.92 (3.80)	Cost per year for clinic with FMS 6.80 (1.85), without FMS 3.16 (0.86)	Cost per year for clinic with FMS 13.03 (3.55), without FMS 0.00	Cost per year for clinic with FMS 266.75 (72.76), without FMS 156.77 (42.76)	Cost per year for clinic with FMS 163.44 (44.58), without FMS 154.64 (42.18)	Cost per year for clinic with FMS 613.83 (167.44), without FMS 443.89 (121.08)	NA	NA	NA	Cost per year for clinic with FMS 1093.88 (298.39), without FMS 786.75 (214.61)

Cost values are reported as MYR (USD average conversion rate per year of that particular financial year); NA–not available.

**Table 6:** Methodological quality of the economic evaluation studies.

	**All Studies**			**Azmi et al. [33]**			**Mustapha et al. [31]**			**Ismail et al. [32]**			**Lim et al. [30]**			**Zhang et al. [37]**			**Fiebert et al. [36]**		
	**Yes**	**P**	**No**	**Yes**	**P**	**No**	**Yes**	**P**	**No**	**Yes**	**P**	**No**	**Yes**	**P**	**No**	**Yes**	**P**	**No**	**Yes**	**P**	**No**
1. Was a clear definition of the illness given?	12	0	0	Yes			Yes			Yes			Yes			Yes			Yes		
2. Were epidemiological sources carefully described?	10	0	2	Yes			Yes			Yes					No	Yes			Yes		
3. Were costs sufficiently disaggregated?	6	3	3	Yes			Yes					No			No			No	Yes		
4. Were activity data appropriately assessed?	7	2	3	Yes			Yes				P		Yes				P				No
5. Were the sources of all cost values analytically described?	6	4	2	Yes				P		Yes				P			P		Yes		
6. Were unit costs appropriately valued?	12	0	0	Yes			Yes			Yes			Yes			Yes			Yes		
7. Were the methods adopted carefully explained?	9	3	0	Yes			Yes			Yes				P		Yes				P	
8. Were costs discounted?	4	0	8			No			No			No			No			No			No
9. Were the major assumptions tested in a sensitivity analysis?	4	0	8			No	Yes					No			No			No			No
10. Was the presentation of study results consistent with the methodology of the study?	10	2	0	Yes			Yes			Yes				P		Yes			Yes		
11. Total score by study *	80	14	26	8	0	2	8	1	1	6	1	3	3	3	4	5	2	3	6	1	3
	**Al-Haddad et al. [29]**			**Amin et al. [27]**			**Ezat et al. [28]**			**Annemans et al. [35]**			**Rohana et al. [26]**			**Seng et al. [34]**					
	**Yes**	**P**	**No**	**Yes**	**P**	**No**	**Yes**	**P**	**No**	**Yes**	**P**	**No**	**Yes**	**P**	**No**	**Yes**	**P**	**No**			
1. Was a clear definition of the illness given?	Yes			Yes			Yes			Yes			Yes			Yes					
2. Were epidemiological sources carefully described?			No	Yes			Yes			Yes			Yes			Yes					
3. Were costs sufficiently disaggregated?	Yes				P		Yes				P		Yes				P				
4. Were activity data appropriately assessed?	Yes			Yes					No	Yes			Yes					No			
5. Were the sources of all cost values analytically described?	Yes					No	Yes				P		Yes					No			
6. Were unit costs appropriately valued?	Yes			Yes			Yes			Yes			Yes			Yes					
7. Were the methods adopted carefully explained?	Yes				P		Yes			Yes			Yes			Yes					
8. Were costs discounted?	Yes					No			No	Yes			Yes			Yes					
9. Were the major assumptions tested in a sensitivity analysis?	Yes					No			No	Yes			Yes					No			
10. Was the presentation of study results consistent with the methodology of the study?	Yes				P		Yes			Yes			Yes			Yes					
11. Total score by study *	9	0	1	4	3	3	7	0	3	8	2	0	10	0	0	6	1	3			

* Total score by study was the sum of all answers: partially (P), yes (1), no (0).
